# The Efficacy of a Nurse-Led Disease Management Program in Improving the Quality of Life for Patients with Chronic Kidney Disease: A Meta-Analysis

**DOI:** 10.1371/journal.pone.0155890

**Published:** 2016-05-18

**Authors:** Chong-Cheng Chen, Yi Chen, Xia Liu, Yue Wen, Deng-Yan Ma, Yue-Yang Huang, Li Pu, Yong-Shu Diao, Kun Yang

**Affiliations:** 1 Department of Nephrology, West China Hospital, Sichuan University, No.37 Guo Xue Xiang, Chengdu 610041, Sichuan Province, P.R.China; 2 Department of Gastrointestinal Surgery, West China Hospital, Sichuan University, No.37 Guo Xue Xiang, Chengdu 610041, Sichuan Province, P.R.China; The University of Tokyo, JAPAN

## Abstract

**Background:**

The impacts of nurse-led disease management programs on the quality of life for patients with chronic kidney disease have not been extensively studied. Furthermore, results of the existing related studies are inconsistent. The focus of the proposed meta-analysis is to evaluate the efficacy of nurse-led disease management programs in improving the quality of life for patients with chronic kidney disease.

**Methods:**

Literature survey was performed to identify the eligible studies from PubMed, Current Nursing and Allied Health Literature, and Cochrane Central Register of Controlled Trials with predefined terms. The outcome measured was quality of life. This meta-analysis was conducted in line with recommendations from the preferred reporting items for systematic reviews and meta-analyses.

**Results:**

Eight studies comprising a total of 1520 patients were included in this meta-analysis, with 766 patients assigned to the nurse-led disease management program. Nurse-led disease management improved the quality of life in terms of symptoms, sleep, staff encouragement, pain, general health perception, energy/fatigue, overall health and mental component summary when evaluated 6 weeks after the beginning of intervention. When evaluated 12 weeks later, the quality of life in terms of symptoms, sleep, staff encouragement, energy/fatigue, and physical component summary was improved. Stratified by the modalities of dialysis, similar results of pooled analyses were observed for patients with peritoneal dialysis or hemodialysis, compared with the overall analyses. The results of sensitivity analyses were the same as the primary analyses. The symmetric funnel plot suggested that the possibility of potential publication bias was relatively low.

**Conclusion:**

Nurse-led disease management program seems effective to improve some parameters of quality of life for patients with chronic kidney disease. However, the seemingly promising results should be cautiously interpreted and generalized and still need to be confirmed through well-designed large-scale prospective randomized controlled trials.

## Introduction

Characterized by gradually impaired renal function and almost irreversible progression to the end-stage renal disease (ESRD), chronic kidney disease (CKD) has become a major public health problem today, approximately affecting 11–13% of the general population [1–3]. Although renal replacement treatments could prolong life expectancy, the long-term illness and related treatments have a strong impact on the physical, psychological and social well-being of patients [4]. Also, deterioration in physical and mental conditions of CKD patients in the course of disease leads to a worse prognosis.

CKD patients typically suffer from frequent readmission and severely limited daily activities, which causes enormously heavy burdens on patients and their families [5] and consequently the poor quality of life (QoL). Therefore, the goals of treatment for CKD patients should not only aim to control symptoms, decrease complication rate, and delay disease progression but also to improve QoL [6].

In the current medical model, a single approach is not adequate to treat the patients with chronic disease [7]. In order to improve QoL, integrated comprehensive care is required, especially for patients under poor control of disease [8,9]. For this purpose, a multidisciplinary nurse-led disease management program, which collaborates, evaluates, and designs services and treatments to satisfy patients’ health needs, is deemed more suitable and necessary [10]. It has been reported that such programs are beneficial for patients with chronic diseases including diabetes, chronic obstructive pulmonary disease, coronary heart disease and *etc*., in terms of physical and mental functioning, self-care ability, self-satisfaction, treatment adherence and QoL [11–14]. Moreover, a nurse-led case management program has been proven to yield positive effects for patients with CKD, including preventing avoidable readmissions, improving health status and reducing the care burden of families [10,15]. Although most available studies have not shown benefits of GFR decline or reduction of other cardiovascular risk factors, it has been reported to be beneficial in reducing blood pressure, low-density lipoprotein, cholesterol, anemia, proteinuria, and hyperphosphatemia along with the improved compliance to the dietary restriction of salt and fluid intake in patients with CKD [16–19].

Unfortunately, most of the previous studies on nurse-led disease management programs focus mainly on the effectiveness of solving the physical or psychosocial problems. Only few studies have noticed their impacts on QoL, which is an important outcome measure for patients with CKD in terms of overall well-being. Meanwhile, the results of these studies were inconsistent. In several studies, significant improvements of QoL were noted for patients with nurse-led disease management programs compared to the control group [10,14,20–23]. However, some other studies have reported that there was no significant difference in QoL between patients in the nurse-led disease management program and those not in the program [16,17]. Even the significant items of QoL were inconsistent among those studies that have demonstrated positive outcomes [10,14,21]. The small sample size in each of the above-mentioned studies might account for the inconsistency, which is not sufficient to provide valid evidence for the benefits of nurse-led disease management programs. Therefore, the efficacy of a nurse-led disease management program in improving the QoL for CKD patients remains unclear. Accordingly, we performed this meta-analysis to evaluate the efficacy of the nurse-led disease management programs in improving the quality of life for patients with chronic kidney disease.

## Methods

This meta-analysis does not require approval of an ethical committee. In order to guarantee its quality, this meta-analysis was conducted in line with recommendations from the preferred reporting items for systematic reviews and meta-analyses protocols (PRISMA statement) [24].

### Search Strategy and Study Selection

We searched electronic databases of PubMed with the search terms of “chronic kidney disease”, “CKD”, “renal failure”, “renal insufficiency”, “end stage renal disease”, “ESRD”, “peritoneal dialysis”, “hemodialysis”, “nurse”, “nursing” “QoL”, “quality of life”, and “life quality”. Current Nursing and Allied Health Literature (CINAHL), and Cochrane Central Register of Controlled Trials (CENTRAL) were also searched with corresponding search terms. Moreover, the reference lists from relevant articles were screened for eligibility. In order to avoid publication biases, the eligible unpublished grey papers were also considered to be included if known by Prof. Diao YS.

The electronic search was not limited to any date of beginning in order to minimize the biases and achieve the thoroughness of the search [25], but was limited until September, 2015. The language of the publications was limited to English.

### Inclusion and Exclusion Criteria

Only randomized controlled trials (RCTs) which compared the impact of nurse-led disease management program on the QoL with non-nurse-led disease management program were eligible.

Articles included in this study considered CKD patients receiving either hemodialysis or peritoneal dialysis. Each stage of CKD was included and there were no limitations of the values of eGFR or creatinine clearance. There were also no limitations of age, sex, and race. However, the QoL must be reported in the study. The exclusion criteria included articles on: renal transplantation recipients, patients diagnosed with mental illness, patients with difficulty in communication to obtain QoL data, and patients with severe comorbidities, such as New York Heart Association class IV heart failure, severe diabetic foot or lupus erythematosus, and *etc*., which can seriously influence the QoL.

### Selection, Assessment, and Data Extraction

In the process of primary screening, two independent reviewers read the title and abstract of each retrieved article to select studies for further assessment. If the title and abstract suggested that the study can be potentially included, full texts were obtained and further assessed.

The data extraction process was conducted independently by two reviewers. The extracted data included first author, publication year, study design, region of the study, number of included centers as well as items for quality assessment such as methods of randomization, allocation concealment, blinding evaluation, and intention to treat analysis. We also collected detailed information of enrolled patients (sample size of each group, inclusion and exclusion criteria, characteristics of study population, and baseline characteristics comparability), interventions (the detail of intervention in each group, the number and reason of withdrawals and dropouts as well as the characteristics of these patients), and outcomes (e.g.. non-adherence, symptom and complication control, blood chemistry, functional capacity, and QoL).

We assessed the quality of the included studies according to the following items [26]: 1) A truly random allocation method was used; 2) The allocation concealment was performed; 3) The baselines between two groups were comparable; 4) The inclusion and exclusion criteria were described clearly; 5) The blinding evaluation of the outcomes was performed; 6) The number and reason of withdrawals and dropouts in each group were demonstrated, and 7) The intention to treat analysis was used. Each item with ‘yes’ was scored 2, “partly yes” 1.5, “unclear” 1, and “no” 0. Studies with a total score less than 10 were regarded to have high possibilities of biases. Any discrepancies between the two independent reviewers in study searching, data extraction, and quality assessment were discussed and resolved by a third reviewer.

### Outcomes of measurements

QoL was adopted as the outcome measure in the present study in terms of symptoms, effect of kidney disease, burden of kidney disease, work status, cognitive function, quality of social interaction, sexual function, sleep, social support, staff encouragement, patient satisfaction, physical functioning, role-physical, pain, general health perception, emotional wellbeing, role-emotional, social function, energy/fatigue, overall health, physical component summary, and mental component summary.

### Statistical Analysis

Continuous data was calculated as weighted mean differences (WMD) with 95% confidence intervals (CI). The analyses were performed by RevMan 5.3 (downloaded from the Cochrane Collaboration) [27]. When the studies had reported quartile only, the means and standard deviations (SD) were estimated according to Wang X [28]. If the data could not be extracted for meta-analysis, we presented the results in a descriptive and qualitative manner [29]. A P value less than 0.05 was considered as statistically significant. Heterogeneities between trials were tested based on a Chi-squared statistical method. A P value less than 0.10 was considered as statistically significant for heterogeneities. I-square was used to rank the variation of heterogeneity (<25%: low heterogeneity, 25% to 50%: moderate heterogeneity, and > 50%: high heterogeneity) [30]. If heterogeneities existed, random effect model was considered. The subgroup analyses were performed stratified by the different questionnaires for QoL and different study populations (patients with hemodialysis or peritoneal dialysis). In order to exclude poor-quality studies, sensitivity analyses were performed, and only high-quality trials were included [31]. A Funnel plot was generated to check the potential publication bias.

## Results

### Study selection and description of the included studies

**Fig 1:**
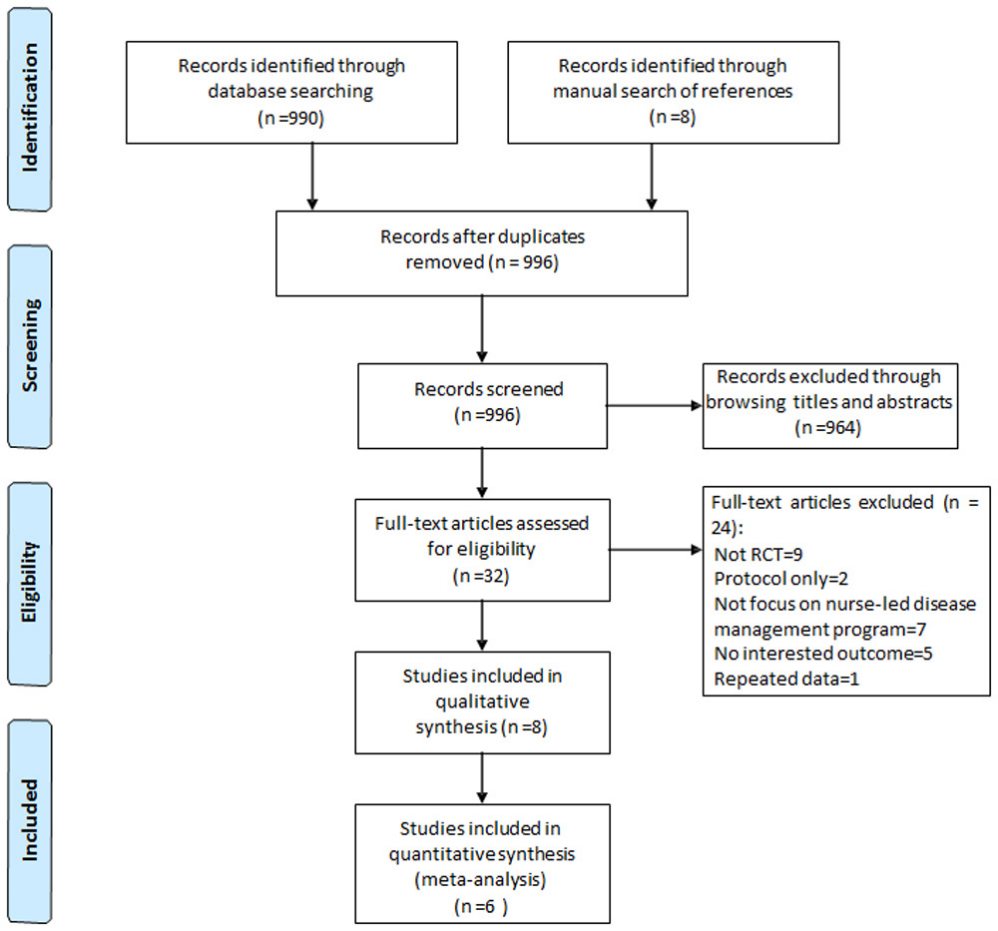
Flow chart for selection of studies. The flowchart of selecting procedure and the exclusive reasons of studies are summarized.

Initially, 998 studies were identified when searched using the predefined search strategy on September, 2015. We selected the related studies according to the inclusion/exclusion criteria described in the Methods section. Then, 966 studies were excluded based on the retrieved titles and abstracts during the primary screening process. We further excluded 24 studies by retrieving and reading the full text of the remaining studies in the secondary selection step. Furthermore, we checked the references of the retrieved studies in order to locate more potentially eligible studies. However, no more studies was found. The flowchart of the search and selection procedure was shown in Fig 1 along with the reasons of exclusion.

Finally, eight RCT studies were selected [10,14,16,17,20–23], and no grey papers were found. The publication years of these studies range from 2007 to 2015. The studies were regionally distributed as follows: two from China main land [21,22], two from Taiwan [20,23], two from Hong Kong [10,14], and two from Netherlands [16,17]. Three studies enrolled patients with peritoneal dialysis [10,14,21], three studies used patients with hemodialysis [20,22,23] and two studies used the estimated creatinine clearance or estimated glomerular filtration rate (eGFR) as the inclusion criteria [16,17]. Six studies applied the questionnaires of the Kidney Disease Quality of Life Questionnaire (KDQOL), KDQOL-SF, KDQOL-36 or SF-36 to evaluate the QoL [10,14,16,17,20–23]. The other two studies applied EQ-5 D or COOP-WONCA charts [16,17]. A total of 1520 patients were available for analysis, with 766 of them treated with nurse-led disease management programs (considered as the intervention group in this meta-analysis). The characteristics and quality assessments of the included trials were listed in Tables 1–3.

**Table 1:** The characteristics of included trials.

Study	Region	Number of centers	Patients	Comparison		Duration of intervention	Outcome measurements	Questionnaire for life quality	Time points of data collection
				Intervention group	Control group				
Lii YC 2007 [20]	Taiwan	2	Hemodialysis patients	Psychosocial intervention (N = 20)	Routine nursing care and a self-care booklet (N = 28)	8 weeks	Self-care self-efficacy; Depression; Quality of life	SF-36	Baseline; 12 weeks later
Wong FK 2010 [14]	Hong Kong	2	Peritoneal dialysis patients	Routine care and the intervention disease management program (N = 49)	Routine care only (N = 49)	6 weeks	Non-adherence; Quality of life; Satisfaction; Symptom and complication control; Health service utilization	KDQOL	Baseline; 6 weeks later; 12 weeks later
Chow SK 2010 [10]	Hong Kong	2	Peritoneal dialysis patients	Comprehensive discharge planning and standardized nurse-initiated telephone follow-up (N = 43)	Routine discharge care (N = 42)	6 weeks	Quality of life	KDQOL-SF	Baseline; 6 weeks later; 12 weeks later
van Zuilen AD 2012 [16]	Netherlands	9	Estimated creatinine clearance between 20 and 70 ml/min	Lifestyle advice and actively address treatment goals (N = 395)	Usual care (N = 393)	1 year	Composite of cardiovascular mortality; Cardiovascular morbidity and overall mortality; Decline of renal function; Change in markers of vascular damage; Change in quality of life	EQ-5 D	Yearly
Scherpbier-de Haan ND 2013 [17]	Netherlands	9	eGFR of <60ml/min/1.73m^2^	Shared care (N = 90)	Routine care (N = 74)	1 year	Lowering of blood pressure; Laboratory biochemical parameters; Functional capacity	COOP-WONCA charts	1 year later
Li J 2014 [21]	China	2	Peritoneal dialysis patients	Comprehensive discharge planning and standardized post-discharge nurse-led telephone support (N = 80)	Routine discharge care (N = 80)	6 weeks	Quality of life; Blood chemistry; Complication control; Health service utilization	KDQOL-SF	Baseline; 6 weeks later; 12 weeks later
Tao X 2015 [22]	China	2	Hemodialysis patients	Incenter group exercise training and nurse case management of home exercise (N = 57)	Group exercise only (N = 56)	12 weeks	Gait speed; 10-repetition sit-to-stand; Quality of life	KDQOL-36	Baseline; 6 weeks later; 12 weeks later
Tsai SH 2015 [23]	Taiwan	1	Hemodialysis patients	Nurse-led breathing training (N = 32)	Waiting for the intervention (N = 32)	4 weeks	Self-reported depressive symptoms; Self-reported sleep quality; Health-related quality of life;	SF-36	Baseline; 6 weeks later

**Table 2:** Details of interventions in included trials.

Study	Details of interventions	
	Intervention group	Control group
Lii YC 2007 [20]	The treatment consisted of eight group sessions, once a week, for two hours. The program had four components, including cognitive behavioral therapy aimed at self-management and coping strategies for stress and depression, restructuring thought patterns and beliefs, stress management, and health education focused on psychosocial skill of self-care strategies.	Routine nursing care and a self-care booklet normally provided by the unit.
Wong FK 2010 [14]	Before the patient was discharged, the renal nurses conducted an initial assessment with the patient. Then, the nurses would make phone calls to the patient every week for 6 weeks. The first call was initiated by the renal nurses within 72 h after the discharge. The subsequent calls were made by the general nurses every week for 4 weeks, including reinforcing appropriate behaviors, identifying potential complications and needs, and reviewing the mutual goal-setting. The final call to review the health goals was finished by the renal nurses.	Instructions on medications and basic health advice
Chow SK 2010 [10]	A comprehensive assessment and individualized education program were conducted by the nurse case manager prior to discharge. After discharge, nurse case managers began telephone-call with patients weekly for six consecutive weeks. The first call was conducted within 72 hours after discharge. In the follow-up calls, the nurse checked and reinforced the patient’s behaviors in achieving the objectives, identified new and potential complications and needs and maintained a sustained relationship with the patient. The community nurses conducted scheduled home visits and reported to the case manager after each home visit.	Routine discharge care, including providing a telephone hotline service, and distributing self-help printed materials on maintaining healthy lifestyles and a reminder to attend the outpatient clinic.
van Zuilen AD 2012 [16]	A nurse practitioner, supervised by a qualified nephrologist, actively pursued lifestyle intervention (physical activity, nutritional counseling, weight reduction, and smoking cessation), the use of specified mandatory medication and the implementation of current guidelines. The nurse practitioner checked regularly whether treatment goals have been achieved and adjust treatment accordingly.	Usual care
Scherpbier-de Haan ND 2013 [17]	The multifaceted intervention consisted of the training of professionals, structured care by nurse practitioners, and the opportunity to ask advice from a nephrology team. The nurse practitioner saw patients every 3 months for a 20-minute consultation. Patients and nurse practitioners decided together which treatment goals were to be prioritized. Nurse practitioners could, if necessary, consult a nephrology team in a protected digital environment.	Routine care
Li J 2014 [21]	A comprehensive assessment and individualized education program were conducted by the nurse case manager prior to discharge. After discharge, nurse case managers began telephone-call with patients weekly for six consecutive weeks. The first call was conducted within 72 hours after discharge. The content of each call was guided by the protocol and the specific problems identified in the predischarge assessment. Any problems the patient encountered were discussed, and some appropriate suggestions were given if necessary.	Routine discharge care, including explaining matters which is needed attention to the patients, providing a telephone hotline service, and distributing self-help printed materials on maintaining healthy lifestyles and a reminder to attend the outpatient clinic.
Tao X 2015 [22]	The center-based group exercise training was delivered weekly for 6 consecutive weeks to a group of four to six patients before dialysis sessions. Each session lasted approximately 20 minutes. The exercise consisted of flexibility and strength exercises. Nurse—patient clinical interview sessions which focused on patient education, barrier identification and solving, mutual goal setting, exercise prescription and exercise monitoring were offered weekly for the first 6 weeks and biweekly for the following 6 weeks. The first session lasted for about 20–30 minutes, and each follow-up session for around 15 minutes. Along with flexibility and strength exercises, the patients were advised to initiate home exercise, including engaging in aerobic exercises.	Center-based group exercise training only
Tsai SH 2015 [23]	Breathing training was carried out twice weekly, for a total of eight sessions. In the first session, participants received individualized coaching of the breathing techniques for 10 minutes by the nurse. Then the participants listened to 10 minutes of prerecorded instruction on breathing techniques, followed by the breathing practice for 20 minutes. During the remaining seven sessions, the participants only listened to the prerecorded voice while practicing breathing for 30 minutes. The nurse supervised each practice session and ensured the efficacy of participants.	The participants were told that they were waiting for the available space for intervention. The participants received four weeks of breathing training after the posttest measurements.

**Table 3:** The quality of included randomized trials.

Study	Truly random	Concealed allocation	Baseline features	Eligibility criteria	Blinding assessment	Loss to follow-up	Intension to treat	Study quality scores^#^
Lii YC 2007 [20]	Yes	Yes	Yes	Yes	Yes	Yes	No	12
Wong FK 2010 [14]	Yes	Unclear	Partly Yes*	Yes	Yes	Yes	No	10.5
Chow SK 2010 [10]	Yes	Unclear	Yes	Yes	Unclear	Yes	No	10
van Zuilen AD 2012 [16]	Yes	Unclear	Partly Yes*	Yes	Yes	Yes	Yes	12.5
Scherpbier-de Haan ND 2013 [17]	Unclear	Unclear	Partly Yes*	Yes	Unclear	Yes	No	8.5
Li J 2014 [21]	Yes	Unclear	Yes	Yes	Unclear	Yes	No	10
Tao X 2015 [22]	Yes	Yes	Yes	Yes	Yes	Yes	Yes	14
Tsai SH 2015 [23]	Yes	Yes	Partly Yes*	Yes	Yes	Yes	No	11.5

*: There was one item of baseline characteristics being not balanced between the two groups.

^#^: The maximum quality score is 14.

### QoL

**Fig 2:**
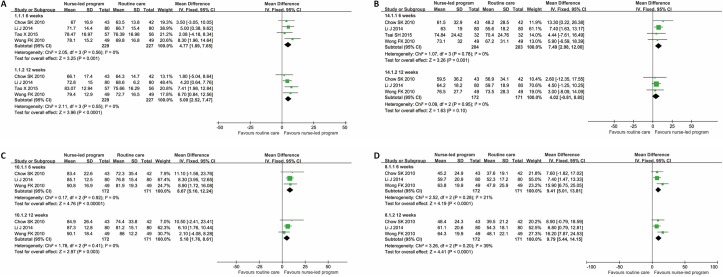
Forest plots of QoL in terms of symptoms, pain, staff encouragement and sleep. A: symptoms; B: pain; C: staff encouragement; D: sleep. The 95% confidence interval (CI) of mean difference for each study is represented by a horizontal line and the point estimate is represented by a square. The size of the square corresponds to the weight of the study in the meta-analysis. The 95% CI for pooled estimates is represented by a diamond. Data for a fixed-effects model are shown as there was no statistical heterogeneity. df = degrees of freedom; I^2^ = percentage of the total variation across studies due to heterogeneity; IV = Inverse Variance; Z = test of overall treatment effect.

**Fig 3:**
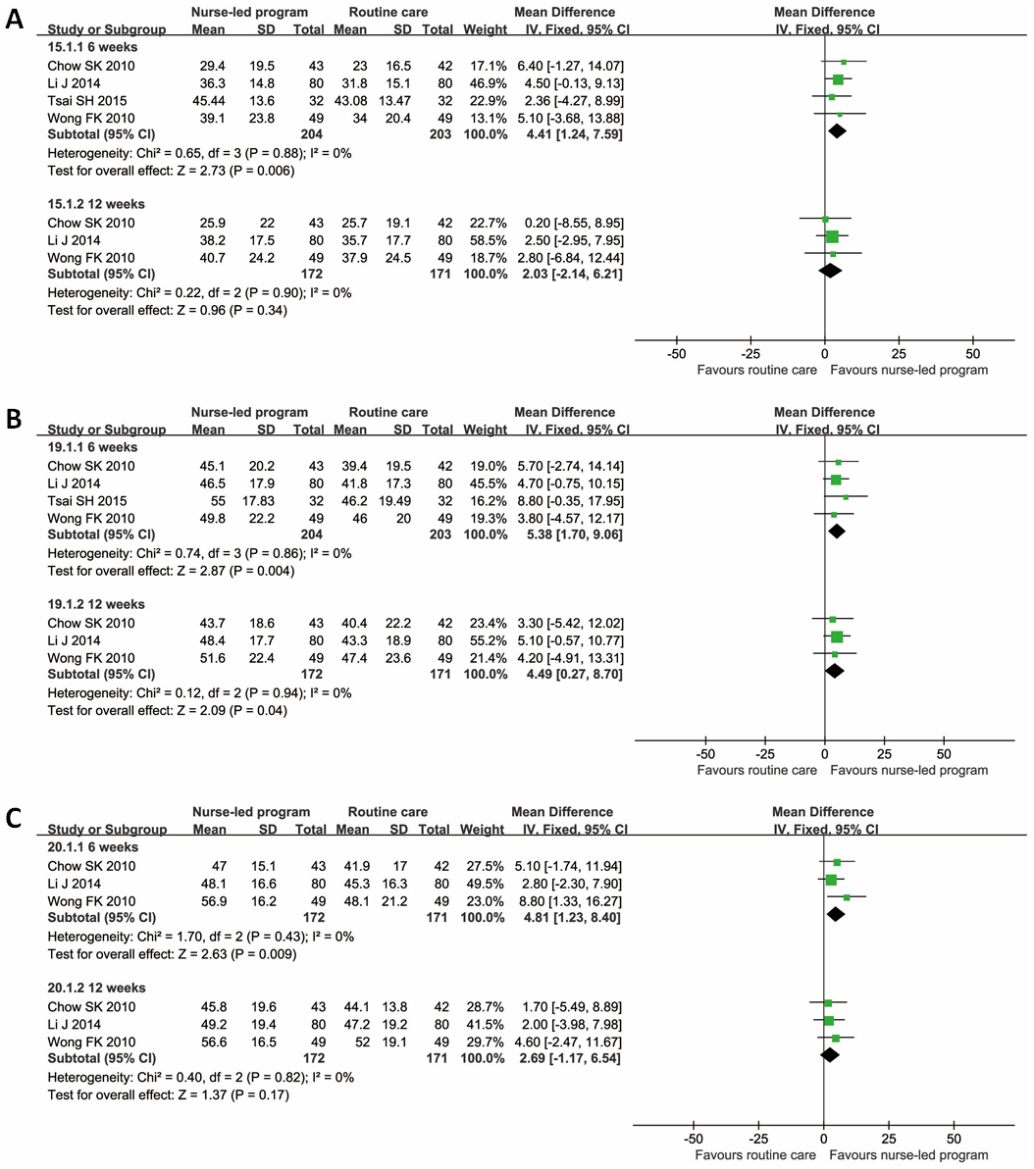
Forest plots of QoL in terms of general health perception, energy/fatigue and overall health. A: general health perception; B: energy/fatigue; C: overall health. The 95% confidence interval (CI) of mean difference for each study is represented by a horizontal line and the point estimate is represented by a square. The size of the square corresponds to the weight of the study in the meta-analysis. The 95% CI for pooled estimates is represented by a diamond. Data for a fixed-effects model are shown as there was no statistical heterogeneity. df = degrees of freedom; I^2^ = percentage of the total variation across studies due to heterogeneity; IV = Inverse Variance; Z = test of overall treatment effect.

Because KDQOL, KDQOL-SF, KDQOL-36 and SF-36 questionnaires are similar and share the same components [32,33], we integrated these questionnaires to perform the meta-analyses. The results of pooled analysis showed that the nurse-led disease management program could indeed improve the QoL in terms of symptoms, sleep, staff encouragement, pain, general health perception, energy/fatigue, overall health, and mental component summary, when evaluated 6 weeks after the beginning of intervention. When evaluated 12 weeks later, the QoL in terms of symptoms, sleep, staff encouragement, energy/fatigue, and physical component summary was improved. Sleep is an item of QoL and was affected more than the others at 6 weeks and 12 weeks. It seemed that the intervention group had a marginally better mental component summary than the control group at the end of 12 weeks, but the difference was not statistically significant (Table 4, Figs 2 and 3).

**Table 4:** Outcomes of meta-analysis of life quality.

Items	Number of studies	Intervention group (N)	Control group (N)	Weighted mean difference [95% CI]	P-value for effect size	I square	P-value for heterogeneity	Effect model
Symptom								
6 weeks	4	229	227	4.77 [1.89, 7.65]	0.001	0	0.56	Fixed
12 weeks	4	229	227	5.00 [2.52, 7.47]	<0.0001	0	0.55	Fixed
Effect of kidney disease								
6 weeks	4	229	227	0.66 [-2.58, 3.90]	0.69	0	0.78	Fixed
12 weeks	4	229	227	1.03 [-2.14, 4.20]	0.52	0	1.00	Fixed
Burden of kidney disease								
6 weeks	4	229	227	-1.38 [-4.27, 1.52]	0.35	0	0.86	Fixed
12 weeks	4	229	227	-0.18 [-3.29, 2.94]	0.91	0	0.54	Fixed
Work status								
6 weeks	3	172	171	1.60 [-1.53, 4.73]	0.32	0.26	0.26	Fixed
12 weeks	3	172	171	1.54 [-1.56, 4.65]	0.33	0.54	0.11	Fixed
Cognitive function								
6 weeks	3	172	171	0.98 [-2.96, 4.91]	0.63	0.43	0.17	Fixed
12 weeks	3	172	171	-2.67 [-6.58, 1.25]	0.18	0	0.39	Fixed
Quality of social interaction								
6 weeks	3	172	171	2.17 [-1.56, 5.90]	0.25	0	0.95	Fixed
12 weeks	3	172	171	2.28 [-1.38, 5.94]	0.22	0	0.99	Fixed
Sexual function								
6 weeks	3	172	171	5.05 [-12.36, 22.46]	0.57	0.93	<0.00001	Random
12 weeks	3	172	171	-0.66 [-10.81, 9.49]	0.90	0.80	0.007	Random
Sleep								
6 weeks	3	172	171	9.41 [5.01, 13.81]	<0.0001	0.21	0.28	Fixed
12 weeks	3	172	171	9.79 [5.44, 14.15]	<0.0001	0.39	0.20	Fixed
Social support								
6 weeks	3	172	171	2.64 [-1.20, 6.48]	0.18	0	0.91	Fixed
12 weeks	3	172	171	1.50 [-2.19, 5.19]	0.43	0	0.68	Fixed
Staff encouragement								
6 weeks	3	172	171	8.67 [5.10, 12.24]	<0.00001	0	0.92	Fixed
12 weeks	3	172	171	5.18 [1.76, 8.61]	0.003	0	0.41	Fixed
Patients satisfaction								
6 weeks	3	172	171	0.91 [-2.27, 4.08]	0.58	0.13	0.32	Fixed
12 weeks	3	172	171	3.71 [-3.69, 11.10]	0.33	0.78	0.01	Random
Physical functioning								
6 weeks	4	204	203	1.93 [-2.00, 5.87]	0.34	0	0.68	Fixed
12 weeks	3	172	171	2.01 [-1.52, 5.53]	0.26	0	0.90	Fixed
Role-physical								
6 weeks	4	204	203	-0.09 [-4.54, 4.36]	0.97	0	0.52	Fixed
12 weeks	3	172	171	-0.37 [-4.51, 3.77]	0.86	0.04	0.35	Fixed
Pain								
6 weeks	4	204	203	7.49 [2.98, 12.00]	0.001	0	0.78	Fixed
12 weeks	3	172	171	4.02 [-0.81, 8.85]	0.10	0	0.95	Fixed
General health perception								
6 weeks	4	204	203	4.41 [1.24, 7.59]	0.006	0	0.88	Fixed
12 weeks	3	172	171	2.03 [-2.14, 6.21]	0.34	0	0.90	Fixed
Emotional wellbeing								
6 weeks	4	204	203	2.68 [-1.05, 6.40]	0.16	0	0.93	Fixed
12 weeks	3	172	171	2.06 [-2.12, 6.24]	0.33	0	0.88	Fixed
Role-emotional								
6 weeks	4	204	203	0.60 [-3.70, 4.89]	0.79	0.42	0.16	Fixed
12 weeks	3	172	171	-0.43 [-4.98, 4.12]	0.85	0	0.97	Fixed
Social function								
6 weeks	4	204	203	1.07 [-3.08, 5.21]	0.61	0.41	0.17	Fixed
12 weeks	3	172	171	-0.90 [-5.66, 3.87]	0.71	0	0.70	Fixed
Energy/fatigue								
6 weeks	4	204	203	5.38 [1.70, 9.06]	0.004	0	0.86	Fixed
12 weeks	3	172	171	4.49 [0.27, 8.70]	0.04	0	0.94	Fixed
Overall health								
6 weeks	3	172	171	4.81 [1.23, 8.40]	0.009	0	0.43	Fixed
12 weeks	3	172	171	2.69 [-1.17, 6.54]	0.17	0	0.82	Fixed
Physical component summary								
6 weeks	2	89	88	-0.39 [-3.18, 2.39]	0.78	0	0.47	Fixed
12 weeks	2	77	84	2.70 [0.07, 5.34]	0.04	0	0.87	Fixed
Mental component summary								
6 weeks	2	89	88	3.12 [0.07, 6.17]	0.05	0.37	0.21	Fixed
12 weeks	2	77	84	3.16 [-0.37, 6.68]	0.08	0	0.94	Fixed

In addition, we performed the subgroup analyses stratified by the modalities of dialysis. Compared with the overall analyses, the same results of pooled analyses were observed for patients with peritoneal dialysis. As for the patients with hemodialysis, the pooled results of life quality in terms of physical component summary and mental component summary were the same as the overall analyses. Only 1 included study with respect to the other items, including symptom, pain, general health, role-emotional, and energy/fatigue, was identified in the hemodialysis subgroup and turned out to be infeasible.

For those studies which were not included in the meta-analysis, we had listed their results as follows:

van Zuilen et al enrolled 788 patients and found that although gradual improvement of life quality by EQ-5D could be observed 1 year later in both intervention and control groups, there was no significant difference between the two groups [16].

Scherpbier-de Haan et al included 163 patients for analysis and reported that there was no significant difference in terms of overall health, daily activities, feelings, physical fitness, social activities, and change in health between the intervention group and control group after 1 year [17].

### Sensitivity analysis

When we re-performed the meta-analyses using high-quality studies only in sensitivity analyses, the results remained the same as the primary analyses.

### Funnel plot

**Fig 4:**
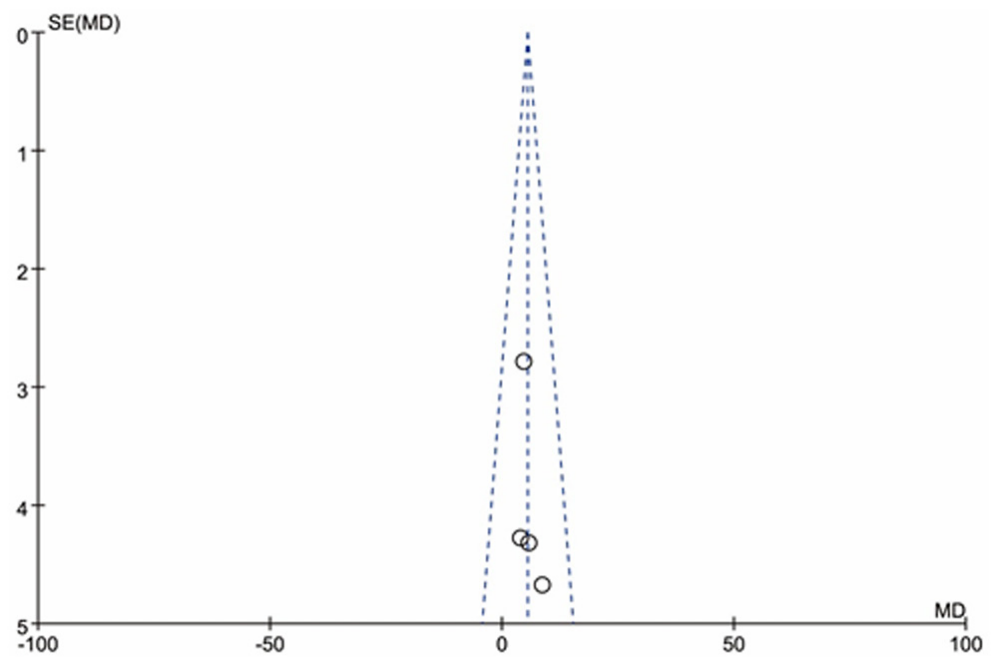
Funnel plot of QoL in terms of energy/fatigue.

The symmetric funnel plot was created in meta-analysis of QoL (See Fig 4). Possibilities of potential publication bias was considered relatively low.

## Discussion

CKD, especially ESRD, represents a major public disease burden worldwide [1–3]. Patients with CKD are confront with the life-long physical, psychological, and social problems [4]. A nurse-led disease management program is essentially a humanization nursing cares with a primary objective of dealing with concerns and needs of patients [10]. It was beneficial to patients with CKD in terms of preventing avoidable readmissions, improving health status, and reducing the care burden of families [10,15]. However, few studies had investigated the impact of a nurse-led disease management program on the QoL of patients with CKD and reported inconsistent outcomes.

In this study, the reasons why only QoL was selected as the outcome measure are listed as follows. Firstly, only few studies focused on changes of QoL with intervention of nurse-led disease management programs. In contrast, the benefits of such programs to address physical or psychosocial problems of CKD patients have been well established [18,19,34]. Secondly, various and heterogeneous outcome measurement parameters about improvement of symptoms, control of renal function, or change of blood chemistry, were used in different studies. It is difficult to integrate them in a single meta-analysis. Thirdly, the improvements of physical or psychosocial problems would result in the change of QoL eventually. Finally, while biological and physiological factors could not be addressed solely by nursing interventions, it has the capacity to impact the life quality of patients [10].

Briefly, our results showed that nurse-led disease management programs could improve the QoL in terms of symptoms, sleep, pain, staff encouragement, energy/fatigue, mental component summary, and physical component summary, which are interactive rather than independent. These programs might take effect in several ways. Firstly, post-discharge nurse-led follow-up could encourage patients and their families to make life style adjustments [10,21,22]. For example, nurse-led disease management programs could assist patients to exercise properly, which was effective in improving the physical function and health self-perception, as well as in alleviating symptoms such as fatigue, sleep disturbances, anxiety, depression, and pain [22, 35–38]. Furthermore, it has been reported that a nurse-led breathing training program could significantly alleviate depressive symptoms, reduce perceived role limitation, and improve the overall mental health component in patients with hemodialysis [23]. Next, our results have showed that nurse-led disease management programs could improve QoL by modifying symptoms, e.g., reducing sleep disturbance, depression, and pain. Sleep quality is an important indicator of health-related QoL. Depression and sleep disturbances often influence each other mutually and generate a vicious circle [39]. Long-term sleep disturbances could lead to anxiety, depression, increased pain frequency, and eventually lower QoL [35,40]. Meanwhile, depression could influence a patient’s mental health, cause somatization symptoms, disturb sleep, and impair functional capacity and social function of CKD patients [41]. Nurse-led disease management programs could provide targeted behavioral and environmental practices to intervene sleep disorder and depression at an early stage, which contributes to improved QoL. Moreover, the prevalence of pain in patients with CKD is usually underestimated. Alleviating the symptom of pain represents a large part of patients’ demand [42]. The reduction in pain could result in less limitation of daily activities and better social functioning and emotional wellbeing. Thirdly, nurse-led disease management programs could encourage the patients to confront their disease in a positive manner and guide them to overcome the reluctance to take renal replacement therapies, which may subsequently contribute to improving the patients’ perception of well-being and reducing the burden of kidney disease [43]. Fourthly, nurse-led disease management program could promote timely detection and intervention of complications, such as hypertension and anemia, which is helpful for the control of symptoms and problems [17]. Finally, nurse-led disease management program could increase the adherence of patients to the healthcare providers and medications, which promotes the patients to actively participate in establishing and achieving treatment goals [14].

However, there were no significant differences in terms of emotional wellbeing, physical functioning, or cognitive function etc., between the two groups. That may be because some physiological factors and comorbid conditions cannot be addressed solely through these programs [44,45]. Furthermore, comorbidity-related re-hospitalization and inadequate home-bound social networks restraint caused by the combination of CKD itself and the dialysis regimen may also negatively affect QoL [10,46]. The third reason was possibly related to the limited sample size and follow-up period of available studies. In our results, there were some variances of results at different time points, which may also be due to fading subjective perceptions or potentially type II error.

Also, there are some limitations of this meta-analysis. Firstly, the included researches for analyzing were limited and regional distribution of these studies is restricted to only the Far East and the Netherlands. More researches need to be investigated to check whether this is generalizable to other countries. In addition, the sample sizes for the pooled meta-analyses were relatively small, adding to the uncertainty of generalizability of these studies. Secondly, some data was obtained through indirect methods, such as some means and standard deviations from the median and interquartile range, which can impair the accuracy of our study to some extent. Thirdly, program design and patient selection in different studies are inconsistent due to the lack of established standardized intervention regimens. The methodological quality of the studies and the questionnaires used to evaluate the effects also varied, which partly explains why the results are controversial. Therefore, our results should be interpreted and generalized cautiously because of the relatively high heterogeneity of the data. Fourthly, the QoL is a kind of subjective data, which is influenced by many factors such as the capacity of understanding or communication and prone to be biased. Therefore, the results are less convincing compared with the objective results (e.g., laboratory test parameters). Fifthly, another potential limitation of the meta-analysis could be overlapped parameters given the use of the specific subscales, as well as the component scores. Finally, the results of pooled meta-analyses lacked the long-term follow-up data. Therefore, the sustained duration of the positive effect after completing the nurse-led disease management program is still unknown. As shown in the results, two included studies have tested the QoL after 1 year and found there had been no significant difference in QoL between the intervention group and control group [16,17]. Although the above limitations exist, we have made an effort to minimize the probability of biases by developing a detailed protocol, performing a cautious search, using objective methods for study selection, data extraction and analysis, and performing the subgroup analyses and sensitivity analyses.

## Conclusion

Nurse-led disease management program seems effective to improve some parameters of quality of life for patients with chronic kidney disease. However, the seemingly promising results should be cautiously interpreted and generalized and still need to be confirmed through well-designed large-scale prospective randomized controlled trials.

## Supporting Information

S1 Checklist PRISMA 2009 checklist for the manuscript. (DOC)

## Abbreviations

CENTRAL: Cochrane Central Register of Controlled Trials
CI: Confidence intervals
CINAHL: Current Nursing and Allied Health Literature
CKD: Chronic kidney disease
eGFR: Estimated glomerular filtration rate
ESRD: End-stage renal disease
PRISMA: Preferred reporting items for systematic reviews and meta-analyses
QoL: Quality of life
RCTs: Randomized controlled trials
SD: Standard deviations
WMD: Weighted mean differences
